# Anti-Microbial Activities of Mussel-Derived Recombinant Proteins against Gram-Negative Bacteria

**DOI:** 10.3390/antibiotics13030239

**Published:** 2024-03-05

**Authors:** Dong Yun Kim, You Bin Oh, Je Seon Park, Yu-Hong Min, Min Chul Park

**Affiliations:** 1 College of Pharmacy, Inje Institute of Pharmaceutical Sciences and Research, Inje University, Gimhae 50832, Republic of Korea; kdy9903@gmail.com; 2 Department of Pharmaceutical Engineering, Inje University, Gimhae 50832, Republic of Korea; dhdbqls1208@gmail.com (Y.B.O.); dolphin3922@naver.com (J.S.P.); 3 College of Health and Welfare, Daegu Hanny University, Gyeongsan 38610, Republic of Korea; yhmin@dhu.ac.kr

**Keywords:** recombinant anti-microbial protein, mussel adhesive protein, Gram-negative bacteria, thermal stability

## Abstract

Many anti-microbial peptides (AMPs) and pro-apoptotic peptides are considered as novel anti-microbial agents, distinguished by their different characteristics. Nevertheless, AMPs exhibit certain limitations, including poor stability and potential toxicity, which hinder their suitability for applications in pharmaceutics and medical devices. In this study, we used recombinant mussel adhesive protein (MAP) as a robust scaffold to overcome these limitations associated with AMPs. Mussel adhesive protein fused with functional peptides (MAP-FPs) was used to evaluate anti-microbial activities, minimal inhibitory concentration (MIC), and time-kill kinetics (TKK) assays against six of bacteria strains. MAP and MAP-FPs were proved to have an anti-microbial effect with MIC of 4 or 8 µM against only Gram-negative bacteria strains. All tested MAP-FPs killed four different Gram-negative bacteria strains within 180 min. Especially, MAP-FP-2 and -5 killed three Gram-negative bacteria strain, including *E. coli*, *S. typhimurium*, and *K. pneumoniae*, within 10 min. A cytotoxicity study using Vero and HEK293T cells indicated the safety of MAP and MAP-FP-2 and -3. Thermal stability of MAP-FP-2 was also validated by HPLC analysis at an accelerated condition for 4 weeks. This study identified that MAP-FPs have novel anti-microbial activity, inhibiting the growth and rapidly killing Gram-negative bacteria strains with high thermal stability and safety.

## 1. Introduction

Infectious diseases, such as SARS-CoV-2, among other viruses, have gained major attention owing to large-scale pandemics, which can severely affect human quality of life and be exacerbated by antibiotic resistance [1,2]. A great need exists for effective antivirals and antimicrobials to impede the transmission of infectious diseases [2,3]. Antiviral and antimicrobial agents are commonly applied on safeguarding materials ranging from consumer goods to medical devices to mitigate the risk of public outbreaks. Although many substances exhibit robust antiviral or antimicrobial activities, a prevailing concern is their unfavorable therapeutic index and resistance [4,5]. Throughout prokaryotes, plants, and vertebrates, antimicrobial peptides (AMPs) are important biomolecules participating in the innate immune response and host defense against infections [6,7,8]. AMPs (typical length of <200 amino acid residues) are secreted by host cells and act as bacteriostatic or bactericidal agents [9]. They often exhibit a distinctive composition featuring cationic and hydrophobic residues that represent amphiphilic structures. Based on their compositional and structural characteristics, AMPs interact with and disrupt the bacterial plasma membranes bearing negative charges in their outer layers [10,11]. Given their diversity and potential as antimicrobial agents, active research is being conducted on the use of AMPs in the therapeutic, medical, and consumer industries. Some AMPs with strong and broad-spectrum antimicrobial activities have been developed for use as antimicrobial substances and antibiotics. FDA-approved AMP-derived antibiotics are used to treat various diseases, depending on their specific characteristics and antimicrobial spectrum. For example, daptomycin, a cyclic lipopeptide sourced from *Streptomyces roseosporus*, is used to treat *Staphylococcus aureus*-associated skin infections [12,13]. Vancomycin, a glycopeptide originating from *Amycolatopsis orientalis*, plays a pivotal role in combatting life-threatening Gram-positive bacterial infections, such as methicillin-resistant *S. aureus* (MRSA) [14]. Nonetheless, the practical application of AMPs is limited by their half-life, cytotoxicity, and production costs [15,16]. Representative AMP-derived antibiotics, including daptomycin, vancomycin, and telavancin, are susceptible to rapid structural degradation owing to instability and high cytotoxicity when administered at high doses, which is often required in clinical treatment. These limitations can be overcome by using recombinant proteins derived from natural materials as scaffolds.

Mussels have a remarkable ability to adhere to surfaces with great stability, even under harsh conditions, such as high pressures and salinity. MAPs are characterized by good biocompatibility and biodegradability, making them useful scaffolds for recombinant proteins [17,18,19,20]. Among the recombinant MAP scaffold proteins, fp-151 (derived from *Mytilus edulis*) is composed of six mussel foot protein 1 decapeptide repeats (fp1) at each mussel terminus of foot protein 5 (fp5) and commonly employed for its high yield, easy purification, and cost-effective manufacturing [21]. Extracellular-matrix-derived peptide-fused MAPs can further be used to regulate cell attachment and growth [22,23]. However, the antimicrobial activities of AMP-fused MAPs have not yet been examined. In this study, we constructed AMP-fused MAPs and determined their antimicrobial performance against various bacteria in vitro.

## 2. Results

### 2.1. Recombinant MAP-Fused Functional Peptides (MAP-FPs)

**Figure 1:**
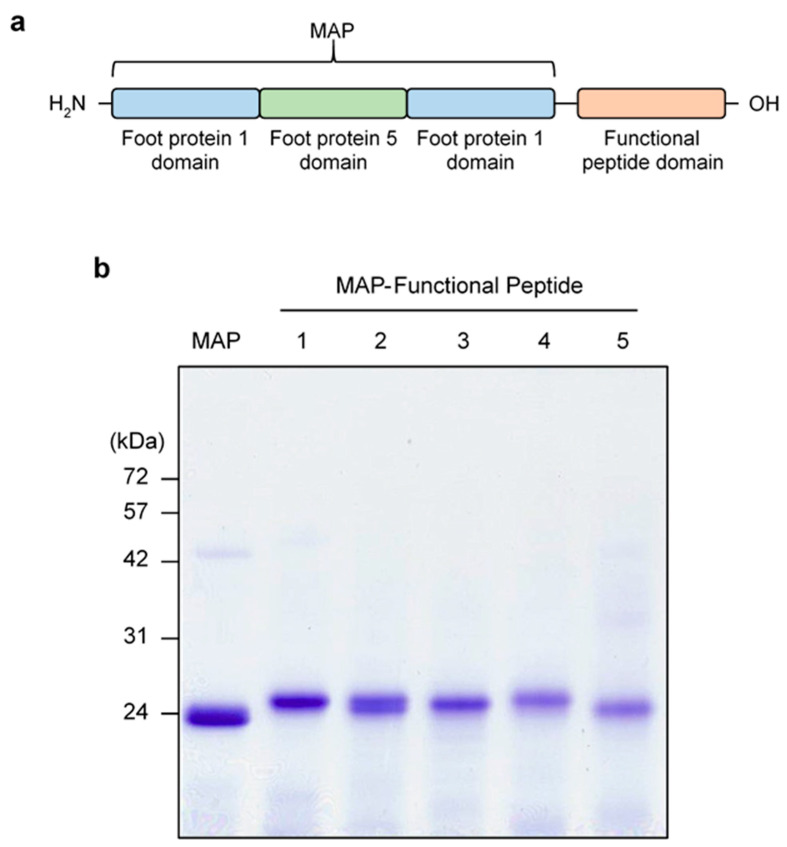
**Schematic representation for MAP-FPs.** (**a**) Schematic diagram of construction for MAP-FPs. (**b**) Electrophoresis analysis of purified recombinant MAP-FPs for purity. Purified recombinant MAP-FPs (5 μg) were loaded with SDS-PAGE and separated using electrophoresis. After electrophoresis, SDS-PAGE was stained with Coomassie blue dye.

Many different proteins, which are derived from living organisms such as metabolites and components of some species, have been tried as biocompatible materials to apply pharmaceutics and medical devices. Although many AMPs were developed in a few decades, there were some limitations, such as low stability and cytotoxicity. To overcome the limitations of AMPs, MAP-derived recombinant protein system was used to give stability and safety to AMPs. Since recombinant mussel adhesive proteins, MAP fp-151, have been developed as biomaterial, having stability and cost-effective manufacturing was used as a scaffold for recombinant AMPs. MAP fp-151 was composed as fp1-fp5-fp1, derived from *Mytilus edulis* [23]. To make MAP-fused functional peptide for anti-microbial application, a number of antibiotic peptides originating from AMPs and proapoptotic peptides were fused to C-terminal of MAP fp-151 (Figure 1a). Anti-microbial-activity-associated peptides, including AKRHHGYKRKFH from anti-microbial histatin 5-derived P-113 (MAP-FP-1) [24], LKKLAKLALAF from anti-cancer peptide (MAP-FP-2) [25], THRPPMWSPVWP from transferrin receptor binding peptide (MAP-FP-3) [26,27], ILRWPWWPWRRK from anti-microbial omiganan (MAP-FP-4) [28,29], and KLAKLAKKLAKLAK from proapoptotic/anti-microbial KLAK peptide (MAP-FP-5) [30,31], were selected for MAP-fused functional peptides (MAP-FPs) (Table 1). Recombinant MAP-FPs were overexpressed and purified as previously described [21]. Since overexpressed recombinant MAP-FPs were aggregated as an inclusion body in *E. coli*, they lost anti-microbial activity and could be purified. Purified recombinant MAP-FPs showed >80% purity, as determined by SDS-PAGE (Figure 1b).

### 2.2. Determination of Minimal Inhibitory Concentrations (MICs) of MAP-FPs

MICs of MAP-FPs and Magainin I were determined against standard laboratory microbial strains, including four Gram-negative and two Gram-positive strains, using the broth microdilution method. The growth inhibitory activities of MAP-FPs were assessed at concentrations of 0.25–8 µM against Gram-negative bacteria, including *Escherichia coli*, *Salmonella typhimurium*, *Klebsiella pneumoniae*, and *Citrobacter freundii*, and Gram-positive bacteria, including *Staphylococcus aureus* and *Bacillus cereus* (Table 2). MAP-FPs and Magainin I at 4–8 µM inhibited the growth of Gram-negative strains. However, neither the MAP-FPs nor Magainin I inhibited the growth of Gram-positive strains. Interestingly, MAP, used as the backbone and adhesive domain for the surface coating, also inhibited the growth of the Gram-negative *E. coli* and *S. typhimurium* strains. Compared to the other MAP-FPs, MAP-FP-2, -4, and -5 showed a two-fold higher growth inhibitory potency against *E. coli* and *S. typhimurium*. These results demonstrate that 4–8 µM of MAP and MAP-FPs could inhibit the growth of susceptible Gram-negative bacteria.

### 2.3. Time-Dependent Microbicidal Activity of MAP-FPs

**Figure 2:**
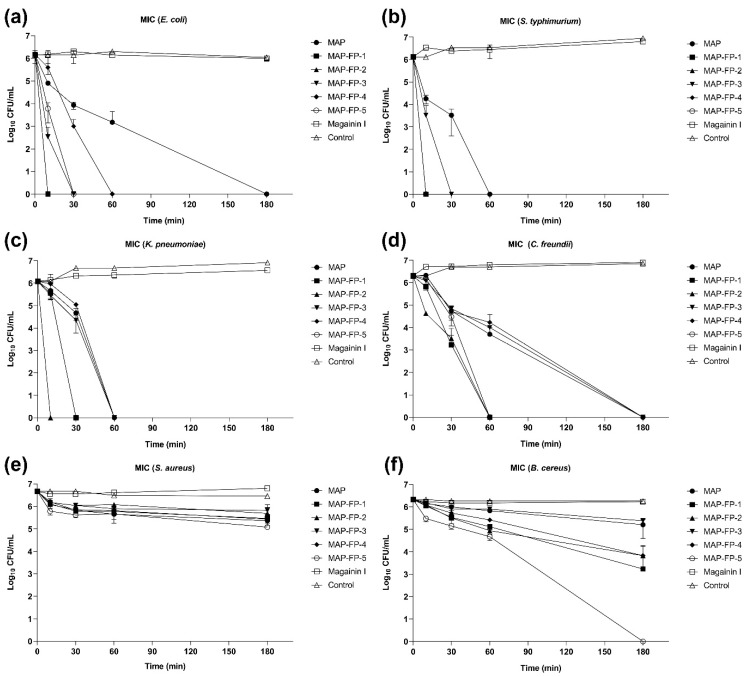
**Time-killing kinetics of MAP-FPs against Gram-negative and -positive bacteria.** Microbials (1.0 × 10^7^ CFU/mL), including *E. coli* (**a**), *S. typhimurium* (**b**), *K. pneumoniae* (**c**), *C. freundii* (**d**), *S. aureus* (**e**), and *B. cereus* (**f**), were treated with 1 × MIC of MAP-FPs and Magainin I in PBS. 1 × MIC stands for 4 µM. After indicated times (10, 30, 60, and 180 min), bacteria were harvested and then spread onto an agar plate. After 18 h of incubation, the CFU was calculated. All data represent the mean value ± standard deviation of three independent experiments.

To check the time-dependent microbicidal activity of MAP-FPs, TKK assay was performed on six microbial strains. TKK of MAP-FPs was investigated using the CFU counting method after indicated times, 0, 10, 30, 60, and 180 min with 1 × MIC of MAP-FPs. Since some MICs have not yet been determined, for all MAP-FPs against Gram-positive bacteria and MAP against *K. pneumoniae* and *C. freundii*, 8 µM was used as 1 × MIC of MAP-FPs and Magainin I. Time-kill kinetics of 1 × MIC MAP and MAP-FPs showed all four Gram-negative bacteria were killed within 180 min, but Magainin I did not (Figure 2a–d). *S. typhimurium* and *K. pneumoniae* were killed within 60 min with MAP and MAP-FPs (Figure 2b,c). Similar to the findings of a previous MIC study, MAP-FP-2 and -5 showed the fastest antimicrobial effects and could kill *E. coli*, *S. typhimurium*, and *K. pneumoniae* within 30 min (Figure 2a–c). Although none of the 1 × MIC MAP-FPs killed *S. aureus* (Figure 2e), MAP-FP-5 killed *B. cereus* within 180 min (Figure 2f). A 1/4 × MIC of MAP-FPs killed all Gram-negative bacteria except *C. freundii* within 180 min (Figure S1a–f). Compared with the TKK of 1 × MIC results, it was observed that even smaller amounts of MAP-FPs also killed susceptible bacteria within 180 min. These results demonstrate the direct microbicidal effects of MAP and MAP-FPs against susceptible Gram-negative bacteria.

### 2.4. Cytotoxicity of the MAP-FPs

To investigate the cytotoxicity of MAP-FPs, Vero, monkey kidney epithelial cells, and HEK293T, human kidney epithelial cells, cell lines were used. Two normal kidney epithelial cells were treated with MAP-FPs in a dose-dependent manner. After 24 h of incubation, cell viability was measured to determine the 50% cytotoxic concentration (CC_50_) of MAP-FPs to normal cells. Although MAP showed good safety even at 12 µM, MAP-FP-1, -4, and -5, derived from P-113, omiganan, and KLA proapoptotic peptide, showed a CC_50_ of 8.3, 5.8, and 7.3 µM, respectively, in only the Vero cell line. Although some MAP-FPs were toxic at high concentrations, MAP-FP-2 and -3 demonstrated good safety at 12 µM (Table 3 and Figure S2a–f).

### 2.5. Thermal Stability of the MAP-FPs

Since MAP-FP-2 exhibited anti-microbial activity without cytotoxicity in normal mammalian cells among MAP-FPs, thermal stability of MAP and MAP-FP-2 was analyzed using RP-HPLC. For thermal stability, MAP and MAP-FP-2 were incubated at 42 °C for 14 and 28 days, and then stability was determined by RP-HPLC. MAPs, incubated at 42 °C, showed 100.02 ± 0.19% and 96.36 ± 0.3% stabilities after 14 and 28 days, respectively. Stabilities of MAP-FP-2 were exhibited as 90.89 ± 1.44% and 91.33 ± 2.6% after 14 and 28 days incubation at 42 °C (Table 4). Although MAP-FP-2 showed a 9% decrease in stability compared to MAP, its stability did not decrease in a time-dependent manner, and its antimicrobial activity was sustained (Table 4). Based on these results, MAP and MAP-FP-2 exhibit strong thermal stability.

## 3. Discussion

There is an urgent global need for new strategies and drugs to treat anti-microbial-resistant bacterial infections. While the presence of antibiotic-resistant strains does not render all existing antibiotics completely useless, it significantly reduces the survival rates of individuals infected with antibiotic-resistant strains. To eliminate antibiotic-resistant bacteria, novel anti-microbial agents should be developed. The traditional antibiotic development pipeline has been unable to address the clinical issues associated with antimicrobial resistance. Thus, the development of novel classes of antibiotics with new targets or modes of action is necessary. New anti-microbial alternatives such as AMP are especially promising because of their potent and broad anti-microbial activity with their slow rate inducing resistance in bacteria [32,33]. In this study, AMPs exhibiting antimicrobial, anticancer, and proapoptotic activities were fused with a biocompatible MAP scaffold to produce a novel antimicrobial MAP-FP, serving as a functional peptide domain. The manufactured MAP and MAP-FPs exhibited strong antimicrobial activities against specific Gram-negative bacteria. Some antibiotics, including aztreonam, cephalosporin, and polymyxin B, also exhibit antimicrobial activities against Gram-negative bacteria. In particular, similar to MAP-FPs, aztreonam did not show anti-microbial activity against Gram-positive bacteria, but only against Gram-negative bacteria. Similar to Gram-negative-specific antibiotic mechanisms, it disrupted the Gram-negative cell wall by binding enriched lipopolysaccharide (LPS) or synthesis enzyme; MAP-FPs are also expected to have a Gram-negative cell wall targeting mechanism [34,35,36].

MAP-FPs not only exhibit unique anti-microbial activity against Gram-negative bacteria but also have favorable therapeutic index and physicochemical properties, such as high thermal stability and adhesiveness. Unlike synthetic adhesives, MAPs possess distinctive properties; mussel filaments (byssus) can attach to inorganic surfaces in the sea without degradation or alteration [37]. This high stability is also reflected in MAP-FPs, which remained structurally stable at 42 °C over 4 weeks, far surpassing the rapid degradation of peptides under similar conditions. This highlights the potential of MAPs as scaffold materials for attaching to inorganic surfaces and incorporating with specific peptides as a functional peptide domain for various applications. Based on these physicochemical properties of MAP-FPs, MAP-FPs possess promise as anti-microbial coating materials against Gram-negative bacteria like as *E. coli*, *S. typhimurium*, and *P. aeruginosa* in the medical devices and consumer goods. Especially, *E. coli* and *P. aeruginosa* are representative bacteria to make biofilm on the surface of medical devices and consumer goods [38,39]. Biofilms, having resistance to anti-microbial agents and host immunity, are the product of microbial cells sticking to each other and adhesive surfaces. Because biofilm is not easily removed by common sterilization and biofilm formation takes some time, biomedical devices need to be coated with an anti-microbial and stable agent to suppress biofilm formation [40]. Since not only MAP-FPs have stable and adhesive properties based on MAP but also have anti-microbial activity based on anti-microbial peptides, MAP-FPs could be good candidates for anti-microbial/biofilm coating agents. Further studies are needed to validate the anti-biofilm activity and adhesiveness to elucidate the practical applications in consumer products and medical devices.

## 4. Materials and Methods

### 4.1. Preparation of Recombinant Proteins

MAP fp-151, cloned into pET-22b(+) vector (Novagen, Darmstadt, Germany), was used as the backbone vector [2]. cDNAs encoding anti-microbial peptides or cell lytic peptides were subcloned into C-terminal of MAP fp-151 at sites *Hind*III and *Xho*I restriction enzymes and expressed in *E. coli* Rosetta (DE3) (Novagen, Darmstadt, Germany) by IPTG (Sigma-Aldrich, St. Louis, MO, USA) induction. MAP-fused functional peptides (MAP-FPs) were purified as previously described [21]. Briefly, induced *E. coli* was lysed using a high-pressure homogenizer. MAP-FPs were extracted using 25% (*v*/*v*) acetic acid from the inclusion body of lysates. Extracts were dialyzed in phosphate-buffered saline (PBS) containing 20% glycerol using dialysis tubing (Sigma-Aldrich, St. Louis, MO, USA). Purified MAP-FPs were freeze-dried and then stored at −80 °C.

### 4.2. Microorganisms

The microorganisms used in this study were *Escherichia coli* (E. coli, ATCC, 25922), *Salmonella typhimurium* (*S. typhimurium*, ATCC, 13311), *Klebsiella pneumoniae* (*K. pneumoniae*, ATCC, 10031), *Citrobacter freundii* (*C. freundii*, ATCC, 6750), *Staphylococcus aureus* (*S. aureus*, ATCC, 29213), and *Bacillus cereus* (*B. cereus*, ATCC, 27348), obtained from the American Type Culture Collection (ATCC, Manassas, VA, USA). The strains were maintained at −80 °C with broth containing 20% glycerol. The broth that was used for the storage of *E. coli*, *S. typhimurium*, *K. pneumoniae*, *C. freundii*, and *B. cereus* in cryogenics was nutrient broth (MB Cell, Seoul, Republic of Korea), while Tryptic soy broth (MB Cell, Seoul, Republic of Korea) was used for *S. aureus*.

### 4.3. Minimal Inhibitory Concentrations (MICs) Determinations

The minimal inhibitory concentrations (MICs) of the MAP-FPs were determined using the broth microdilution method in Cation-adjusted Mueller Hinton Broth (CAMHB, MB Cell, Seoul, Republic of Korea) against all six strains following the guidelines of the Clinical and Laboratory Standard Institute (CLSI). The M7-A9 protocol was used for MIC assay. Briefly, *E. coli*, *S. typhimurium*, *K. pneumoniae*, *C. freundii*, and *B. cereus* were incubated in nutrient broth, and *S. aureus* was incubated in Tryptic soy broth overnight at 37 °C. Then, six strains of bacteria were grown overnight at 37 °C, 150 RPM in the CAMHB and were diluted with CAMHB to achieve 0.5 McFarland turbidity standard (1.5 × 10^8^ CFU/mL, OD_600_: 0.07~0.08) using a microplate reader. Next, a total of 100 μL of the 0.5 McFarland solution was added to 9900 μL of CAMHB (1/100 dilution, 1.5 × 10^6^ CFU/mL). To make final inoculum as 1.5 × 10^5^ CFU/mL, 10 μL of bacterial inoculum containing 1.5 × 10^6^ CFU/mL cells was added into the plate (SPL, Gyeonggi-do, Republic of Korea) wells containing 90 μL of serially diluted peptide samples (final concentrations ranging from 0.25 to 8 µM) and incubated at 37 °C for 18 h. The inhibition of microbial growth was determined by cell counting kit-8 (CCK-8) reagent (Dojindo, Kumamoto, Japan). Incubated broth of each well was diluted 10-fold with PBS using a multi-channel pipette. A volume of 10 μL of CCK-8 reagent was added to each well and incubated for 90 min. The absorbance at 450 nm wavelength was measured using the microplate reader (BioTek Synergy, Winooski, VT, USA, S1LFA). All measurements were performed in triplicate. 

### 4.4. Time-Kill Kinetics (TKK) Assay

The time-kill kinetics (TKK) assay of the MAP-FPs was studied against six different microbial strains. A volume of 670 μL of the 0.5 McFarland turbidity standard solution, having microbials of 1.5 × 10^8^ CFU/mL, was added to 9330 μL of PBS without bacteria to adjust the inoculum concentration, having 1.0 × 10^7^ CFU/mL. Adjusted microbials (1.0 × 10^7^ CFU/mL) were incubated with PBS containing 1 × MIC (4 µM: MAP-FP-2, -4, -5, Magainin I against *E. coli*, *S. typhimurium*; Magainin I against *K. pneumoniae*/8 µM: MAP, MAP-FP-1, -3 against *E. coli*, *S. typhimurium*; MAP, MAP-FPs against *K. pneumoniae*; MAP, MAP-FPs, Magainin I against *C. freundii*, *S. aureus*, and *B. cereus*) and 1/4 × MIC (1 µM: MAP-FP-2, -4, -5, Magainin I against *E. coli*, *S. typhimurium*; Magainin I against *K. pneumoniae*/2 µM: MAP, MAP-FP-1, -3 against *E. coli*, *S. typhimurium*; MAP, MAP-FPs against *K. pneumoniae*; MAP, MAP-FPs, Magainin I against *C. freundii*, *S. aureus*, and *B. cereus*) of MAP and MAP-FPs. Then, incubation of PBS containing MAP-FPs and microbials was performed under the conditions of 37 °C, 150 RPM. After 0, 10, 30, 60, and 180 min, incubated microbials were harvested, serially diluted, and then 100 μL of serially diluted sample containing microbials were spread onto a nutrient agar plate. Agar plates were then further incubated at 37 °C for 24 h. CFUs were determined by counting colonies manually. Non-treated cultured bacteria were used as a control.

### 4.5. Cytotoxicity

Vero (KCLB, 10081) and HEK293T (ATCC, CRL-3216) were obtained from the Korean Cell Line Bank (KCLB, Seoul, Republic of Korea) and ATCC (Manassas, VA, USA), respectively. Vero and HEK293T cells were cultured at 37 °C, 5% of CO_2_ with Roswell Park Memorial Institute (RPMI)-1640 and Dulbecco’s Modified Eagle’s Medium (DMEM) High Glucose (Hyclone, Logan, UT, USA) containing 10% fetal bovine serum (FBS, Hyclone, Logan, UT, USA), 100 Unit/mL penicillin, 100 μg/mL streptomycin, respectively (Hyclone, Logan, UT, USA). Vero and HEK293T (5 × 10^3^/well) cells were seeded in 96-well cell culture plates (SPL, Gyeonggi-do, Republic of Korea) with 10% FBS medium. After 15 h, cells were exposed to media only or media containing various concentration of MAP and MAP-FPs for 24 h. Cytotoxicity was measured using CCK-8 solution following manufacturer’s instructions (Dojindo, Kumamoto, Japan). Briefly, 10 μL of CCK-8 solution was added to cultured media and incubated for 90 min to detect absorbance. The absorbance was measured at 450 nm wavelength using a microplate reader (BioTek Synergy, Winooski, VT, USA). CC_50_ values were calculated using Prism (GraphPad, version 9.1.1).

### 4.6. Thermal Stability Analysis

MAP and MAP-FPs, dissolved in deionized water, were aliquoted and incubated at 42 °C for 2 and 4 weeks (final concentration was 8 µM). After incubation, thermal stability of MAP-FPs was analyzed by reverse-phase high-performance liquid chromatography (RP-HPLC) instrument. RP-HPLC was performed on an Agilent 1100 series HPLC system coupled with a diode array detector (Agilent, Santa Clara, CA, USA, G1315A). A Phenomenex gemini C18 column (250 × 4.6 mm, 5 µm; Phenomenex, 00G-4435-E0, Torrance, CA, USA) protected by a C18 security guard column (SecurityGuard; Phenomenex, Torrance, CA, USA) was used for chromatographic separation at 40 °C. Chromatographic separation was performed in gradient mode with a flow rate of 1 mL/min. Gradient elution, consisting of an organic phase (A, 0.1% trifluoroacetic acid (TFA) in acetonitrile (ACN)) and an aqueous phase (B, 0.1% TFA in water), was changed from 0:100 (A:B, *v*/*v*) to 100:0 (A:B, *v*/*v*) in 50 min. The sample injection volume was 10 μL, and protein identification was made by UV detection at 280 nm. The data were analyzed by ChemStation B.04.03 (Agilent, Santa Clara, CA, USA). All measurements were performed in triplicate. Stability was expressed as the percentage recovery which was calculated from the following equation.

Recovery (%) = purity of analyzed sample / purity of analyzed control (day 0) × 100

### 4.7. Statistical Analysis

Statistical analysis was performed using an unpaired, two-tailed, Student’s *t*-test with GraphPad Prism 9.1.1 (GraphPad, San Diego, CA, USA). The data were presented as mean ± standard deviation. *p* < 0.05 was considered statistically significant.

## Figures and Tables

**Table 1:** Chemical properties of the MAP-fused functional peptides (MAP-FPs).

Peptide	Sequence	Molecular Weight	Hydrophobic Residues (%)	Net Charge at pH 7.0
MAP-FP-1	AKRHHGYKRKFH	1564.82	42	5.27
MAP-FP-2	LKKLAKLALAF	1215.59	27	3.00
MAP-FP-3	THRPPMWSPVWP	1490.75	17	1.09
MAP-FP-4	ILRWPWWPWRRK	1780.16	33	4.00
MAP-FP-5	KLAKLAKKLAKLAK	1524.01	43	6.00
Magainin I	GIGKFLHSAGKFGKAFVGEIMKS	2409.88	30	3.09

**Table 2:** **Minimum inhibitory concentration of MAP-FPs against Gram-negative and -positive bacteria.** Microbials (1.5 × 10^5^ CFU/mL), including *E. coli*, *S. typhimurium*, *K. pneumoniae*, *C. freundii*, *S. aureus*, and *B. cereus*, were incubated with MAP-FPs and Magainin I (concentration range 0.25~8 µM) in CAMHB. After 18 h of incubation, the growth inhibition of microbials was measured using CCK-8 reagent. A volume of 10 μL of CCK-8 reagent was added to each well and incubated plate for 90 min at 37 °C. Absorbance of 450 nm wavelength was determined using microplate reader. Data represent the result of the experiment performed in triplicate. n.d. means not determined. Data are presented as the mean ± standard deviation.

Peptide	Gram-Negative								Gram-Positive			
	*E. coli*		*S. typhimurium*		*K. pneumoniae*		*C. freundii*		*S. aureus*		*B. cereus*	
	MIC (µM)	*p* Value	MIC (µM)	*p* Value	MIC (µM)	*p* Value	MIC (µM)	*p* Value	MIC (µM)	*p* Value	MIC (µM)	*p* Value
MAP	8	<0.0001	8	<0.0001	n. d.	-	n. d.	-	n. d.	-	n. d.	-
MAP-FP-1	8	<0.0001	8	<0.0001	8	<0.0001	8	0.0001	n. d.	-	n. d.	-
MAP-FP-2	4	<0.0001	4	<0.0001	8	0.0001	8	0.0011	n. d.	-	n. d.	-
MAP-FP-3	8	<0.0001	8	<0.0001	8	0.0091	n. d.	-	n. d.	-	n. d.	-
MAP-FP-4	4	<0.0001	4	<0.0001	8	<0.0001	8	<0.0001	n. d.	-	n. d.	-
MAP-FP-5	4	<0.0001	4	0.0001	8	<0.0001	8	<0.0001	n. d.	-	n. d.	-
Magainin	4	<0.0001	4	<0.0001	4	<0.0001	8	<0.0001	n. d.	-	n. d.	-

**Table 3:** **Cytotoxicity of the MAP-FPs in mammalian cells.** Vero and HEK293T cells were used for cytotoxicity of MAP-FPs. Cells (5000 cells/well) were seeded and then treated with different concentration of MAP-FPs for 24 h. After 24 h of incubation, cytotoxicity was determined by CCK-8 solution. A volume of 10 μL of CCK-8 reagent was added to each well and incubated plate for 90 min at 37 °C, 5% CO_2_. Data represent the result of the experiment performed in triplicate.

Peptide	CC_50_ (µM)	
	Vero	HEK293T
MAP	>12	>12
MAP-FP-1	8.3	>12
MAP-FP-2	>12	>12
MAP-FP-3	>12	>12
MAP-FP-4	5.8	>12
MAP-FP-5	7.3	>12

**Table 4:** **Thermal stability analysis of MAP and MAP-FP-2.** MAP and MAP-FP-2, dissolved in deionized water (8 µM), were incubated at 42 °C for 14 and 28 days. After incubation, MAP and MAP-FP-2 were analyzed using RT-HPLC. To check anti-microbial activity, the MIC of incubated MAP and MAP-FP-2 against *E. coli* was determined by the microdilution method.

Day of Storage	MAP		MAP-FP-2	
	Recovery ± RSD	MIC (µM)	Recovery ± RSD	MIC (µM)
0	100.00 ± 0.78	8	100.00 ± 3.61	4
14	100.02 ± 0.19	8	90.89 ± 1.44	4
28	96.36 ± 0.30	8	91.33 ± 2.60	4

## Data Availability

Data are contained within the article and Supplementary Materials.

## Supplementary Materials

The following supporting information can be downloaded at: https://www.mdpi.com/article/10.3390/antibiotics13030239/s1, Figure S1: Time killing kinetics of MAP-FPs 1/4 × MIC against Gram-negative and positive bacteria; Figure S2: Cytotoxicity of the MAP-FPs in Vero and HEK293T cells.
