# Supplementary material for: Anti-Microbial Activities of Mussel-Derived Recombinant Proteins against Gram-Negative Bacteria
Source: Antibiotics (Basel). 2024 Mar 5;13(3):239. doi: 10.3390/antibiotics13030239 (PMC10967471; doi:10.3390/antibiotics13030239)
Supplement: Supplementary file 1 [file antibiotics-13-00239-s001.zip › antibiotics-2883186-supplementary.pdf]

Supporting Information

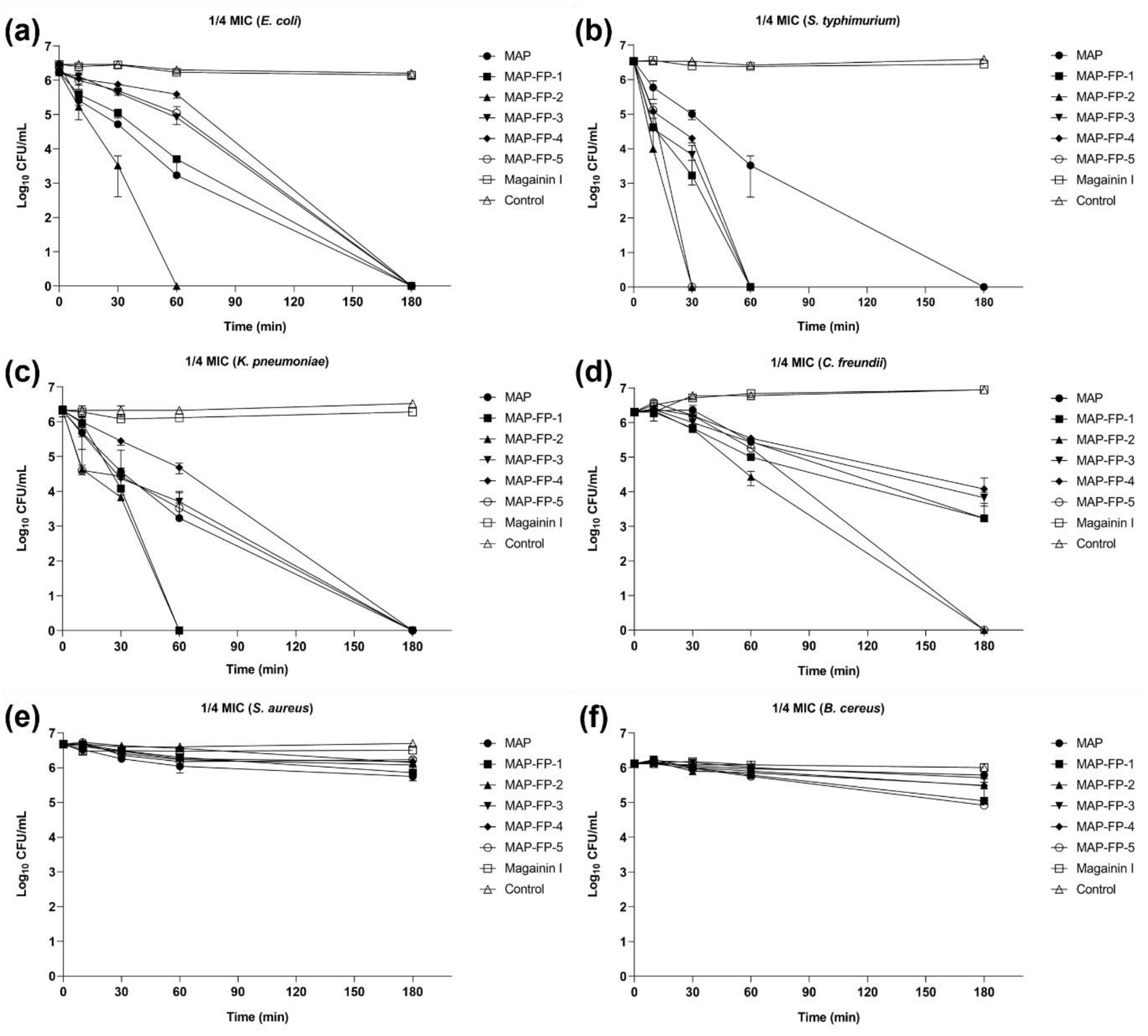

Figure S1. Time killing kinetics of MAP-FPs 1/4×MIC against Gram-negative and positive bacteria. Microbials (1.0×10^7 CFU), including *E. coli* (a), *S. typhimurium* (b), *K. pneumoniae* (c), *C. freundii* (d), *S. aureus* (e), and *B. cereus* (f), were treated with 1/4×MIC of MAP-FPs and Magainin I in PBS. After indicated times (10, 30, 60 and 180 min), bacterial was harvested and then spread into agar-plate. After 18 hr incubation, CFU was calculated by counting the number of colonies. All data represent the mean value ± standard deviation of three independent experiments.

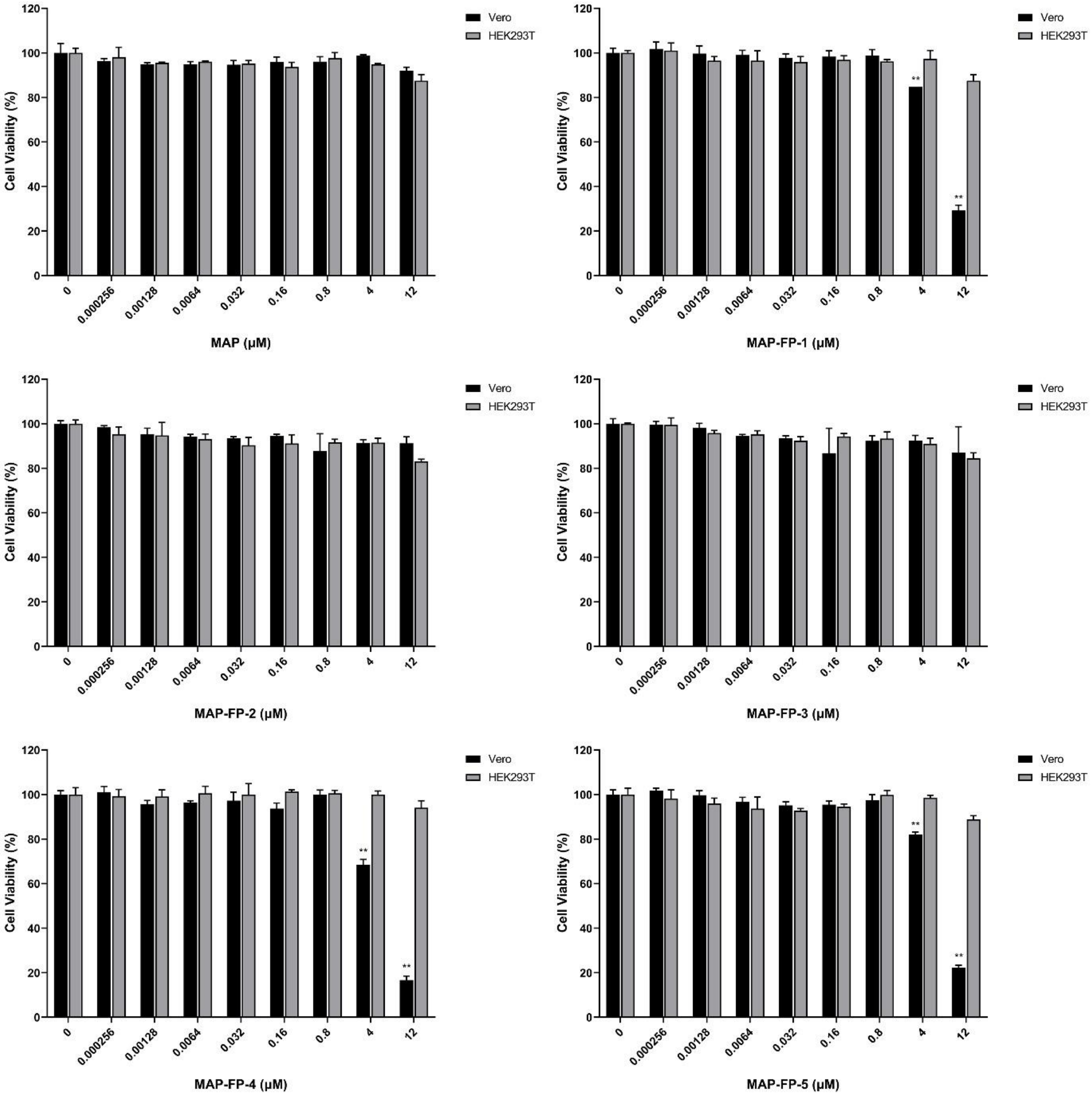

Figure S2. Cytotoxicity of the MAP-FPs in Vero and HEK293T cells.

All data represent the mean value ± standard deviation of three independent experiments. Data are presented as the mean ± standard deviation. *$p < 0.05$, **$p < 0.01$
